# Relationship between Spinal Cord Volume and Spinal Cord Injury due to Spinal Shortening

**DOI:** 10.1371/journal.pone.0127624

**Published:** 2015-05-22

**Authors:** Feng Qiu, Jin-Cheng Yang, Xiang-Yang Ma, Jun-Jie Xu, Qing-Lei Yang, Xin Zhou, Yao-Sheng Xiao, Hai-Sheng Hu, Li-Hui Xia

**Affiliations:** Department of Orthopedics, Guangzhou General Hospital of Guangzhou Military Command, No.111 Liuhua Avenue, P.O. Box 510010, Guangzhou, 510010, People’s Republic of China; Hospital Nacional de Parapléjicos - SESCAM, SPAIN

## Abstract

Vertebral column resection is associated with a risk of spinal cord injury. In the present study, using a goat model, we aimed to investigate the relationship between changes in spinal cord volume and spinal cord injury due to spinal shortening, and to quantify the spinal cord volume per 1-mm height in order to clarify a safe limit for shortening. Vertebral column resection was performed at T10 in 10 goats. The spinal cord was shortened until the somatosensory-evoked potential was decreased by 50% from the baseline amplitude or delayed by 10% relative to the baseline peak latency. A wake-up test was performed, and the goats were observed for two days postoperatively. Magnetic resonance imaging was used to measure the spinal cord volume, T10 height, disc height, osteotomy segment height, and spinal segment height pre- and postoperatively. Two of the 10 goats were excluded, and hence, only data from eight goats were analyzed. The somatosensory-evoked potential of these eight goats demonstrated meaningful changes. With regard to neurologic function, five and three goats were classified as Tarlov grades 5 and 4 at two days postoperatively. The mean shortening distance was 23.6 ± 1.51 mm, which correlated with the d-value (post-pre) of the spinal cord volume per 1-mm height of the osteotomy segment (r = 0.95, p < 0.001) and with the height of the T10 body (r = 0.79, p = 0.02). The mean d-value (post-pre) of the spinal cord volume per 1-mm height of the osteotomy segment was 142.87 ± 0.59 mm^3^ (range, 142.19–143.67 mm^3^). The limit for shortening was approximately 106% of the vertebral height. The mean volumes of the osteotomy and spinal segments did not significantly change after surgery (t = 0.310, p = 0.765 and t = 1.241, p = 0.255, respectively). Thus, our results indicate that the safe limit for shortening can be calculated using the change in spinal cord volume per 1-mm height.

## Introduction

The treatment of severe spinal deformities, namely scoliosis and kyphosis, is complicated by altered anatomy, severe rotation of the vertebrae, and limited flexibility of the spinal column [1, 2]. Vertebral column resection (VCR), which shortens the spinal column, is a generally accepted technique for correcting severe spinal deformities [3–5]. However, spinal cord injury (SCI) may occur in cases of excessive spinal shortening [6–8]. The relationship between the amount of shortening and SCI is important for reducing intra- and postoperative neurologic complications. Kawahara et al. [9] reported that the so-called “dangerous range of shortening” was greater than two-thirds of the vertebrectomy length in a dog model. In addition, Hitesh et al. [10] reported that shortening of ≥104.2% of the height of one vertebral body at the thoracolumbar level induced SCI, whereas shortening of ≤73.8% of the height of one vertebral body did not cause SCI in a pig model. However, the safe limits reported in these previous studies were based on the vertebral body height and not on the spinal cord itself, which may yield different findings in different animals. Moreover, to our knowledge, no previous study has examined the relationship between spinal cord volume (SCV) and SCI in gradual spinal shortening surgery. Hence, in the present study, we aimed to utilize a goat model to investigate the relationship between changes in SCV and SCI due to spinal shortening, and to quantify the SCV per 1-mm height in order to clarify the safe limit for shortening.

## Materials and Methods

### Animals

This study was carried out in strict accordance with the recommendations provided in the guide for the care and use of laboratory animals by the authority of the People’s Republic of China, and the study protocol was approved by the animal ethics committee at Guangzhou General Hospital of Guangzhou Military Command (permit number: 2014–0112), All goats were purchased from Sui Northern experimental animal farms and bred in the animal center of Guangzhou General Hospital of Guangzhou Military Command. Ten goats (age, 24–36 months) with an average body weight of 28 ± 2.21 kg (range, 25–32 kg) were used in this experiment. The study animals underwent fasting for 24 hours prior to anesthesia. General anesthesia was induced in each goat with an injection of 0.15 mL/kg of xylazine hydrochloride and 10 mL of 3% pentobarbital sodium before endotracheal intubation. Anesthesia was maintained by administration of 2–4 mL of 3% pentobarbital sodium, according to the condition of each animal [11]. All experiments were performed under the supervision of veterinarians, and all efforts were made to minimize suffering.

### Surgical preparation and positioning

**Fig 1:**
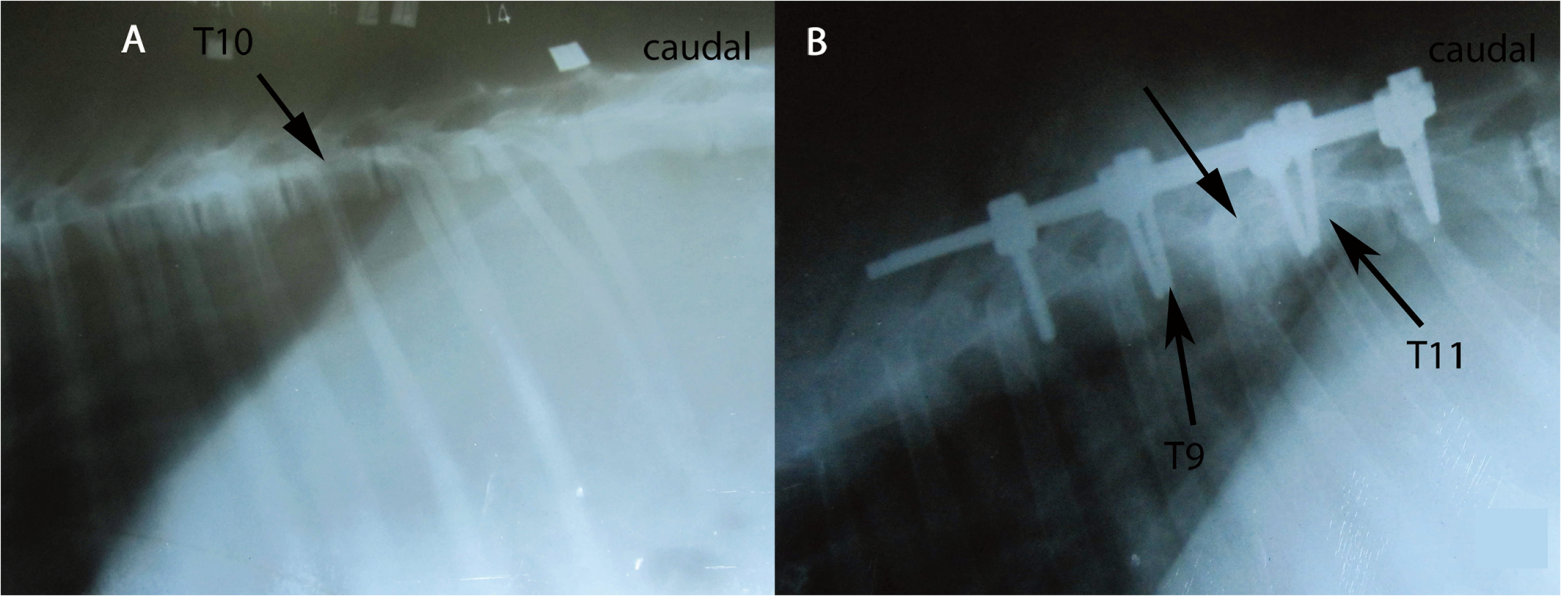
Pre- and postoperative radiographs of T8-12 with the use of a C-arm image intensifier. **(A)** Preoperative radiograph showing T10 marked at the 12^th^ ribs and the surface metal sign. **(B)** Postoperative radiograph showing that the vertebral body of T10 was resected completely and that the internal fixation was satisfactory.

After induction of anesthesia, each goat received an intravenous drip of 5% glucose solution with 240,000 units of gentamicin in order to prevent infection. Each goat was monitored by using a rectal temperature probe and by recording arterial blood pressure. Body temperature was maintained between 36°C and 37°C by using a heating pad when necessary. Arterial blood pressure was maintained by increasing the amount of administered fluid when the pressure decreased. Subsequently, the animals were placed in a prone position on a radiolucent table, and the desired level of surgery (T8-12) was marked using a C-arm image intensifier (Fig 1).

### Preparation for neuromonitoring

The somatosensory-evoked potential (SSEP), which is an electrical response signal in the scalp or spine evoked by stimulating the somatosensory afferent peripheral nerves, was measured by a qualified electrophysiology technician using the Neuron-Spectrum System (Neurosoft, Russia), and the neuromonitoring data were read and analyzed by a neurophysiologist. A stimulating electrode was placed on the left posterior tibial nerve, and a recording electrode was placed at -0.5 cm and 0.2 cm from the intersection of the ear lines and median sagittal line. The placement of the recording electrodes corresponded to the left hindlimb sensory projection area of the cortex. A reference electrode was placed on the hard palate, and pulses of constant stimulation current (20 Hz, 0.2 ms duration) were subsequently delivered. The baseline SSEP amplitude of the first positive wave and baseline peak latency were measured before the experiment. A decrease in the SSEP of more than 50% from the baseline amplitude of the first positive wave or a delay of more than 10% relative to the baseline peak latency was considered an abnormal result [12].

### Surgical procedure

**Fig 2:**
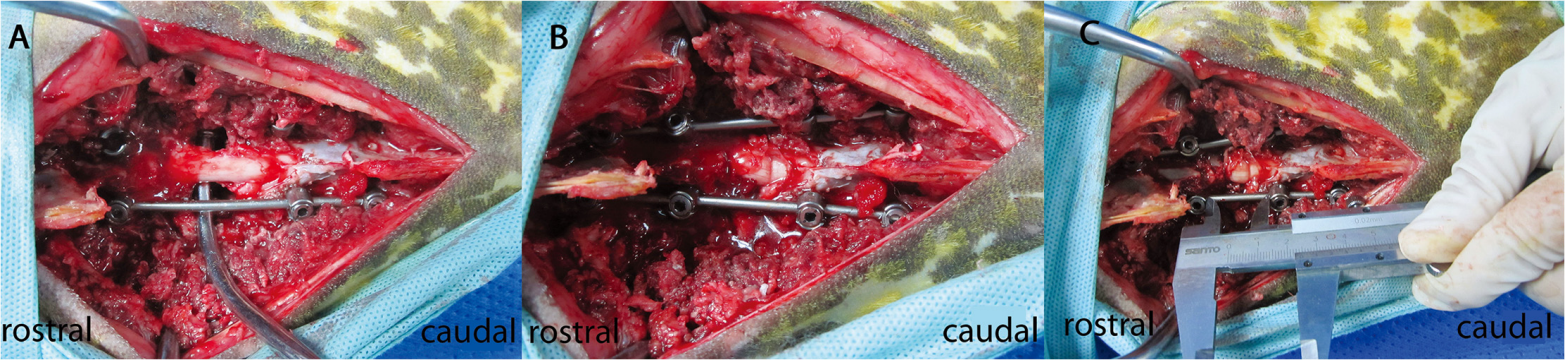
Intraoperative photographs. **(A)** Screws and rods were assembled after completion of the laminectomy and osteotomy, just prior to the shortening procedure. **(B)** After spinal cord shortening, the osteotomy site was stabilized by tightening the pedicle screws on the rods in this position. **(C)** The distances were measured by using a vernier caliper.

**Fig 3:**
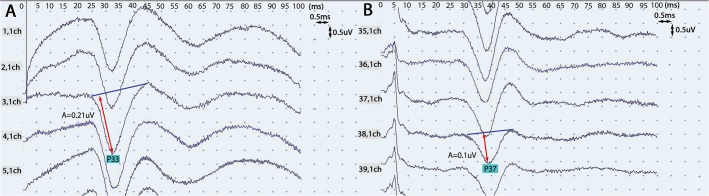
Recordings of somatosensory-evoked potential (SSEP) amplitudes. **(A)** The SSEP under normal conditions. The amplitude of the first positive wave was 0.21 uV and the peak latency was 33 ms. **(B)** The SSEP after spinal cord shortening. The amplitude of the first positive wave was 0.1 uV and the peak latency was 38 ms. The amplitude decreased by 52.4% and the peak latency was delayed by 12% after spinal cord shortening compared with the normal conditions. ch, channel; P, positive; A, amplitude.

A midline longitudinal incision extending from T7-12 was created, and the vertebral laminae were exposed from T8-12. A total laminectomy was performed from T9-11, and 4.5 × 22-mm single-axis pedicle screws were fixed into the vertebrae at T8, T9, T11, and T12. The SSEP was monitored during screw insertion. A rod of suitable length was fixed to the pedicle screws on the right side for temporary fixation. The T10 vertebral body and adjacent discs were resected completely using a curette and osteotome, while carefully avoiding any injury to the dura or spinal cord. After the osteotomy was completed, the rod on the right side was removed, and two rods of suitable length were assembled on each side (Fig 2A). At this time, the SSEP was recorded again, and compared with the baseline amplitude (Fig 3A). Any decrease in the SSEP amplitude during the osteotomy was considered to represent an accidental iatrogenic SCI, and these animals were not included in the analysis.

The spinal column was shortened simultaneously on both sides by using a click-type stopper at 1-mm intervals. After every 3 mm of closure, the procedure was ceased for 60 seconds and the SSEP was recorded. The lengths on the right and left sides were measured using a vernier caliper (Fig 2C), and the mean value of these two measurements was determined. The limit for changes in SCV was defined as the point at which the SSEP was decreased by 50% from the baseline amplitude or delayed by 10% relative to the baseline peak latency (Fig 3B); spinal column shortening was ceased upon reaching this limit. The shortened distance was maintained for 5 minutes after the development of spinal injury. Thereafter, the final distance on each side was measured to determine if there was any recovery of the SSEP. If there was no recovery, the compression was released every 3 mm until recovery of SSEP was observed. Subsequently, the final distance on each side was measured. A wake-up test, which was used to assess the animal’s motor functions, was carried out by discontinuation of the anesthetic to exclude false-negative and false-positive SSEP results. During this test, the movement of the lower extremities and sensory response to mechanical stimulation of the goat were assessed. Subsequently, the goat was anesthetized again. At the final stage of the experiment, the osteotomy site was fixed with a bone graft, and the pedicle screws were tightened at the rods in this position (Fig 2B). Two days after surgery, a postoperative neurologic examination was conducted.

### Postoperative neurologic examination

The spine was shortened by 21.4–25.6 mm in eight goats. Neurologic function was evaluated two days after surgery according to the Tarlov scoring system as follows [13, 14]: grade 0, complete paraplegia with no hind extremity motion; grade 1, minor joint movements; grade 2, major joint movements; grade 3, the animal can stand; grade 4, the animal can walk; and grade 5, the animal can climb a 20°-inclined plane.

### SCV measurement

**Fig 4:**
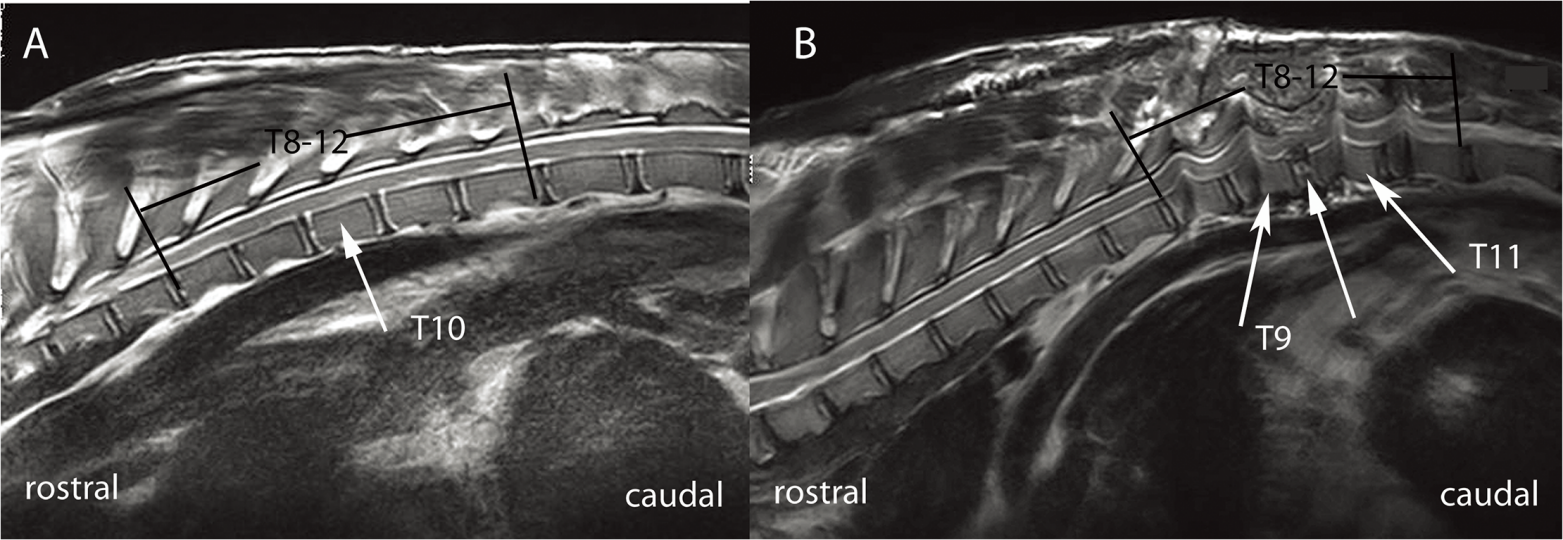
Pre- and postoperative magnetic resonance images (MRIs) of T9-11. **(A)** Preoperative MRI showing a normal signal at the T8-12 spinal cord. **(B)** MRI three days postoperatively showing successful complete resection of the T10 vertebral body and adjacent discs and a normal signal at the T8-12 spinal cord.

**Fig 5:**
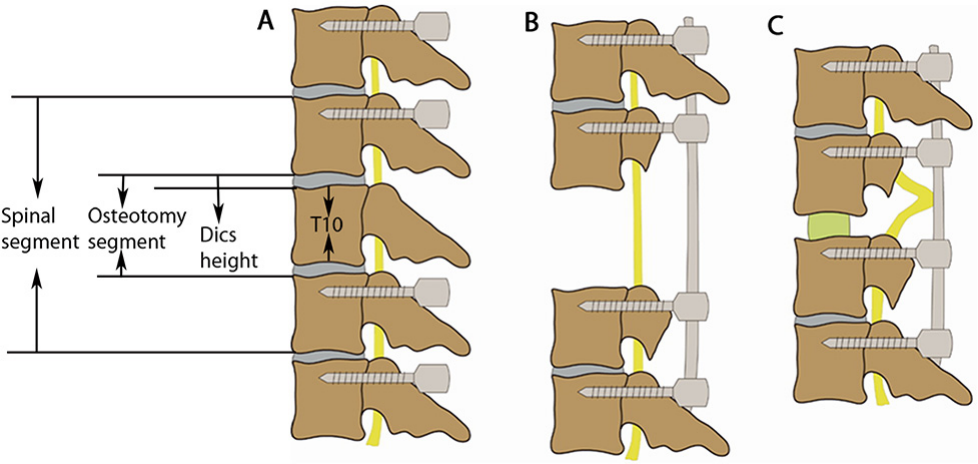
Schematics demonstrating the shortening process. **(A)** T8-12 were exposed, and single-axis pedicle screws were inserted into the vertebrae at T8, T9, T11, and T12. The T10 height (defined as the length from the upper to lower endplates of T10), disc height (defined as the length from the lower endplate of T9 to the upper endplate of T10), osteotomy segment height (defined as the length from the lower endplate of T9 to the upper endplate of T11), and spinal segment height (defined as the length from the upper endplate of T9 to the lower endplate of T11) were measured. **(B)** The T10 vertebral body and adjacent discs were resected completely, and laminectomy of T9-11 was performed. **(C)** The spinal column was shortened, and the spinal cord was buckled.

**Fig 6:**
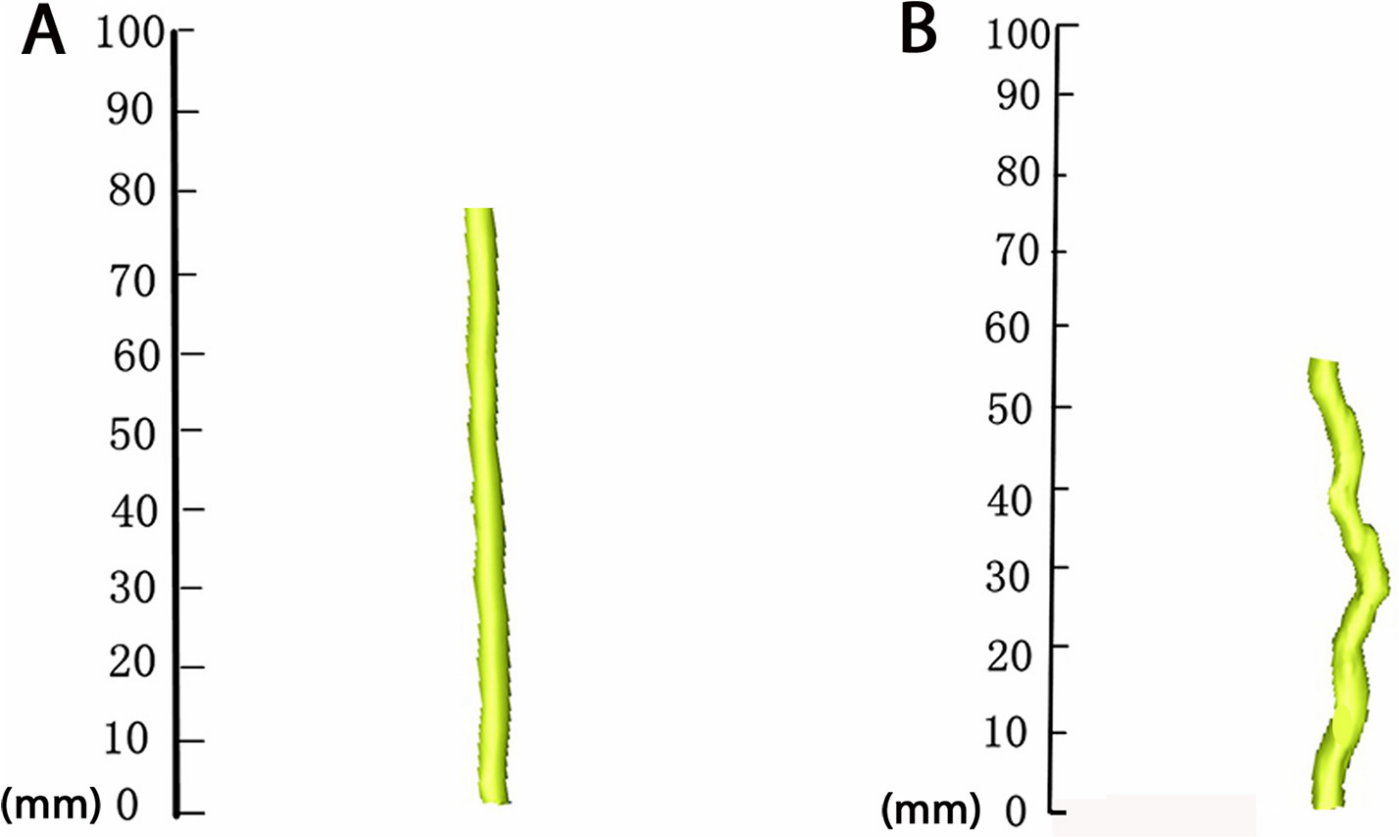
Pre- and postoperative reconstructions of spinal cord volume with the use of Mimics software. **(A)** Preoperative spinal cord volume of T9-11. **(B)** Spinal cord volume of T9-11 at three days postoperatively.

In all animals, magnetic resonance imaging (MRI; Siemens, Germany) of T9-11 (2-mm slice thickness) was conducted preoperatively and three days postoperatively (Fig 4). The MRIs were saved as DICOM 3.0 files and downloaded to a personal computer. The T10 body height, disc height (defined as the length from the lower endplate of T9 to the upper endplate of T10), osteotomy segment height (defined as the length from the lower endplate of T9 to the upper endplate of T11), and spinal segment height (defined as the length from the upper endplate of T9 to the lower endplate of T11) were measured pre- and postoperatively using an MRI workstation (Fig 5). Data of all the regions (DICOM) were imported into Materialise Interactive Medical Image Control System version 15.01 software (Mimics; Materialise, Leuven, Belgium) in order to reconstruct and calculate the SCV. Mimics software can separate the spinal cord, reconstruct it, and use the reconstruction to calculate the SCV. The volumes of the osteotomy and spinal segments were measured pre- and postoperatively (Fig 6). Each value was measured in triplicate, and the mean value of these three measurements was determined and used for the analysis.

### Data analysis

The paired Student’s *t* test was used to compare the pre- and postoperative mean volumes, as well as to compare the mean shortening distance resulting in an SSEP change along with the mean d-value (postoperative minus preoperative value) of the osteotomy segment height. Spearman’s correlation test was used to analyze the relationship between the bilateral shortening distance and other parameters, such as the d-value of the osteotomy segment volume, the d-value of the spinal segment volume, T10 body height, spinal segment height, and disc height. A correlation was considered strong, good, or fair if r = 0.80–0.99, 0.60–0.79, or 0.50–0.59, respectively For all analyses, P ≤ 0.05 was considered significant.

### Preliminary study

**Fig 7:**
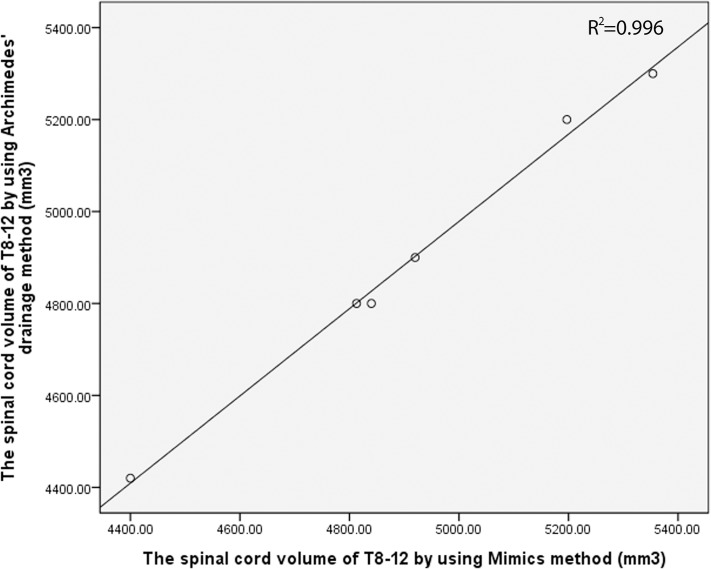
Results of our preliminary study using Archimedes’ drainage method to evaluate the reliability of the measured values. The spinal cord volume of T8-12 was measured using the Mimics method and Archimedes’ drainage method. The measured values of the two methods showed good reliability (R^2^ = 0.996, r = 0.986, p < 0.001).

Prior to this study, a preliminary study was performed to determine the reliability of the measured values. We measured the volume of T8-12 in six goats after the induction of anesthesia by using a previously described method; thereafter, all goats were sacrificed, and the spinal cords were removed and assessed using MRI. The SCV was measured on MRI using Mimics software. In addition, the SCV was also measured using Archimedes’ drainage method. This preliminary study demonstrated that the results measured using these two different methods were consistent (r = 0.986, p < 0.001) (Fig 7). Thus, the method of SCV measurement using MRI and the Mimics software was determined to be reliable.

## Results

Two of the 10 goats were excluded due to changes in the SSEP during the osteotomy or due to postoperative infection. The SSEP of the remaining eight goats (3 female, 5 male) showed meaningful changes; the SSEP of five goats was decreased by 50% from the baseline amplitude and was delayed by 10% relative to the baseline peak latency, whereas the SSEP of the remaining three goats was decreased by 50% from baseline amplitude but was delayed by less than 10% relative to the baseline peak latency. During the wake-up test, all four limbs of all eight goats could move. With regard to neurologic function, five goats were classified as Tarlov grade 5 and three were classified as Tarlov grade 4 at two days after surgery. Data obtained from these eight goats were analyzed.

Intraoperatively, the mean T10 body height, preoperative osteotomy segment height, preoperative spinal segment height, postoperative osteotomy segment height, postoperative spinal segment height, disc height, d-value of the osteotomy segment, and spinal cord shortening distance were 22.1 ± 1.69 mm, 28.9 ± 1.88 mm, 78.7 ± 7.14 mm, 5.4 ± 0.46 mm, 62.0 ± 6.16 mm, 4.1 ± 0.60 mm, 23.6 ± 1.51 mm, and 23.6 ± 1.52 mm, respectively (Table 1). The paired Student’s *t* test used to compare the mean osteotomy segment d-value and mean shortening distance did not reveal any statistical significance (t = -0.076, p = 0.941).

**Table 1:** Lengths of the spinal segments.

Goat No.	Preoperative (mm)			Postoperative (mm)				
	T10 body height	Osteotomy segment	Spinal segment	Osteotomy segment	Spinal segment	Disc height (mm)	d-value of the osteotomy segment (post-pre) (mm)	Shortening distance (mm)
1	24.4	30.4	86	5.4	67	4.1	25	25
2	20	28.1	72.4	5.5	57.9	3.1	22.6	22.3
3	20.9	27	73	5.2	57.3	3	21.8	21.5
4	20.2	26.2	74.8	4.8	59.4	4.6	21.4	21.8
5	22.7	31.8	87.2	6.2	70.8	3.5	25.6	25.4
6	22.7	29.7	77.1	5.4	59.8	2.9	24.3	24.2
7	24.2	30	88	5.8	69.6	3.2	24.2	24.5
8	21.7	28.1	71.3	4.9	54.5	3.4	23.2	24.2
Mean	22.1	28.9	78.7	5.4	62	4.1	23.5	23.6
Std. Dev.	1.69	1.88	7.14	0.46	6.16	0.6	1.51	1.52

Comparison of the mean osteotomy site d-value and mean spinal cord shortening distance showed no statistical significance (t = -0.076, p = 0.941).

The mean pre- and postoperative volumes of the osteotomy and spinal segments were 952.50 ± 71.63 mm^3^ and 950.30 ± 64.57 mm^3^, and 2433.60 ± 138.24 mm^3^ and 2471.87 ± 140.96 mm^3^, respectively (Table 2). No significant differences between the mean pre- and postoperative volumes of the osteotomy segment (paired Student’s *t* test; t = 0.310, p = 0.765) and spinal segment (paired Student’s *t* test; t = 1.241, p = 0.255) were observed.

**Table 2:** Volumes of the spinal cord segments.

Goat No.	Preoperative (mm^3^)		Postoperative (mm^3^)	
	Osteotomy segment	Spinal segment	Osteotomy segment	Spinal segment
1	943.42	2618.16	940.42	2616.20
2	974.00	2504.52	962.30	2498.24
3	915.77	2355.05	900.00	2367.22
4	845.96	2568.43	884.20	2490.20
5	1082.52	2695.13	1080.30	2683.20
6	949.47	2561.71	921.20	2560.10
7	1028.18	2682.60	1021.70	2279.40
8	880.66	2283.20	892.30	2280.40
Mean	952.50	2533.60	950.30	2471.87
Std. Dev.	71.63	138.24	64.57	140.96

The mean preoperative and postoperative volume at the levels of the osteotomy site and spinal segment did not significantly differ (paired t test; osteotomy site: t = 0.310, p = 0.765; spinal segment: t = 1.241, p = 0.255).

The mean pre- and postoperative SCVs per 1-mm height of the osteotomy segment were 33.03 ± 1.25 mm^3^ and 175.91 ± 1.19 mm^3^, respectively. Moreover, the mean pre- and postoperative SCVs per 1-mm height of the spinal segment were 32.29 ± 1.85 mm^3^ and 40.99 ± 1.84 mm^3^, respectively. The mean d-value (post-pre) of the SCV per 1-mm height of the osteotomy segment was 142.87 ± 0.59 mm^3^ (range, 142.19–143.67 mm^3^), whereas the corresponding value of SCV per 1-mm height of the spinal segment was 8.7 ± 0.09 mm^3^ (range, 8.61–8.84 mm^3^), with the osteotomy segment changing more substantially than the spinal segment (Table 3).

**Table 3:** Unit volumes of the spinal cord (1-mm).

Goat No.	Osteotomy segment			Spinal segment		
	Preoperative	Postoperative	d-value (post-pre)	Preoperative	Postoperative	d-value (post-pre)
1	31.03	174.71	143.67	30.44	39.08	8.63
2	34.66	177.09	142.43	34.59	43.26	8.66
3	33.92	176.11	142.19	32.26	41.10	8.84
4	32.54	174.88	142.34	34.45	43.09	8.64
5	33.54	177.21	143.67	30.23	38.95	8.72
6	32.12	175.00	142.88	33.67	42.28	8.61
7	34.24	177.44	143.20	30.22	38.88	8.66
8	32.22	174.82	142.60	32.48	41.29	8.81
Mean	33.03	175.91	142.87	32.29	40.99	8.70
Std. Dev.	1.25	1.19	0.59	1.85	1.84	0.09

Spearman’s correlation test demonstrated a strong correlation between the bilateral shortening distance with the d-value (post-pre) of SCV per 1-mm height of the osteotomy segment (r = 0.95, p < 0.001) and a good correlation with T10 body height (r = 0.79, p = 0.02), whereas no correlation with the d-value (post-pre) of SCV per 1-mm height of the spinal segment was observed (r = -0.26, p = 0.53) (Table 4). Although the spinal segment height and disc height showed good correlations with the shortening distance (r = 0.60 and 0.61, respectively), no statistical significance was observed (p = 0.10 and 0.21, respectively).

**Table 4:** Correlations between morphometric factors and distraction distance.

Morphometric factor	R Value (p-value)*
d-value per 1-mm height of osteotomy segment	0.95 (<0.001)
d-value per 1-mm height of spinal segment	-0.26 (0.53)
T10 body height	0.79 (0.02)
Spinal segment height	0.62 (0.10)
Disc height	0.61 (0.21)

*Spearman’s correlation analysis

## Discussion

Although various osteotomy techniques can be used to correct severe spinal deformities, VCR is the only effective technique to manage severe rigid spinal deformities caused by coronal or sagittal decompensation [15–18]. However, several studies have reported neurologic complications in an average of 14.3% (range, 1.2–17.1%) of VCR cases [3, 6, 18–20]. Such complications may be related to excessive shortening of the spinal cord. The current study investigated the effects of changes in SCV due to spinal shortening during posterior VCR on spinal cord function in eight goats in order to determine the safe limit for spinal shortening.

This experiment utilized young goats aged between 24 and 36 months. The goat spine is similar to the human spine in many aspects, including the structure and biomechanics of the thoracic and lumbar vertebrae [21, 22]. There are 12 dorsal and 6–8 lumbar vertebrae in goats. The spinal cord terminates at the L6 level in goats; however, in humans, it terminates near the L1-2 level. In this study, we chose T10 as the site of VCR, which matches the T9-10 level in humans; the surgical level was located precisely by using the 12th rib of the goat as a marker.

SSEP can be utilized to continuously evaluate sensory neural pathways during surgery, without obstructing the surgeon, even when muscle relaxants are employed intraoperatively [23, 24]. However, relatively high rates of false-negative and false-positive results have been reported [25]. Some of the influencing factors, including hypothermia, hypotension, and inhalation anesthesia, can be avoided [26]. In this study, the body temperature and arterial blood pressure of each goat were monitored to eliminate the effects of low body temperature and blood pressure instability on the conduction velocity. In addition, intravenous anesthesia was used to reduce the influence of inhalation anesthesia on SSEP. Further, in this study, similar to in the study by Strahm et al [27], no anesthesia drugs were given during the spinal shortening in order to ensure the accuracy of detecting SSEP. Finally, the functional status of the spinal cord was evaluated by using the wake-up test after confirming the presence of SCI based on the SSEP changes, in order to reduce the risk of false-negative and false-positive SSEP results [28, 29].

Mimics software is an image-processing tool that connects two-dimensional image data with three-dimensional image engineering. It is widely used in the clinical setting because of its powerful three-dimensional imaging technology and its ability to compute irregular volumes [30, 31]. By using the software configuration function according to a certain value of regional growth, the spinal cord can be easily separated from the surrounding tissue, including the cerebrospinal fluid, so as to accurately calculate the SCV. However, there is no previous report on the use of the Mimics software to measure SCV. Conversely, the Archimedes drainage method has been widely used to measure the volume of irregular objects, by measuring the discharge water volume when an irregular object is totally immersed in water. Thus, the SCV can be accurately calculated by this method by using a fine graduated cylinder. Our preliminary study showed that the results using the Archimedes’ drainage method and Mimics software correlated well (r = 0.986, p < 0.001), and confirmed that the method of SCV measurement on MRI by the Mimics software was reliable.

In this study, when the spinal column was shortened, SCI was defined as the occurrence of decreased amplitude or delayed peak latency of the SSEP. The mean shortening distance was 23.6 ± 1.55 mm; it strongly correlated with the d-value (post-pre) of SCV per 1-mm height of the osteotomy segment and showed a good correlation with T10 body height. The maximum value of the change in SCV per 1-mm height was based on the osteotomy segment, and its safe limit was found to be 142.75 ± 0.68 mm^3^. The safe limit for shortening was approximately 106% of the vertebral height, and the volumes of the spinal segment and osteotomy segments were not significantly changed after surgery. If the preoperative SCV per 1-mm height of the osteotomy segment is known, its corresponding postoperative value can be calculated. Thus, the safe limit for shortening can be calculated preoperatively. In addition, several studies have reported the use of computer-aided design rapid prototyping to preoperatively simulate the operation [30, 32]. Combining computer-aided design prototyping and calculation of the shortening distance preoperatively may help develop a better osteotomy scheme as a means to improve the success of surgery and to reduce the risk of neurologic complications.

Kawahara et al. [9] reported that shortening by more than two-thirds of the vertebral height caused compression and spinal cord deformity due to buckling of the dura in a dog model, while Hitesh et al. [10] reported that SCI was induced upon shortening by 104% of the vertebral height in a pig model. Different results may be seen in different animals, and it has been speculated that this may be largely related to the laminectomy length. In Kawahara’s study, laminectomy was performed from the caudal region of the upper vertebra to the cranial region of the lower vertebra, whereas total laminectomy was performed from the upper vertebra through the lower vertebra in Hitesh’s study. In this study, the osteotomy site was T10, and total laminectomy was performed from T9 through T11. Furthermore, a sheep cadaveric study [33] reported that, during full-length shortening, the mean kink of the spine in the sagittal plane was 92.4° for two-level hemilaminectomy of T11 and T13, 24.6° for complete laminectomy of T11 with hemilaminectomy of T13, and 20.2° for two-level complete laminectomy. The authors reported that it was possible to avert kinking of the spine by applying the proper laminectomy technique for full-length shortening. In this study, we determined the safe limit for shortening by using the SCV per 1-mm height of the osteotomy segment. However, SCI may result from local changes in SCV per 1-mm height even before this safe limit is reached. Of note, the calculated safe limit was based on total laminectomy of T9-11; thus, the morphology should be carefully observed during the actual operation. On the other hand, the previous study described the extent of spinal shortening in relation to the mean body height of the relative vertebrae. However, altered anatomy, such as hemivertebrae or block vertebrae, is often observed at the osteotomy site in cases of severe spinal deformity. Such altered anatomy may lead to alterations of the spinal cord length, spinal morphology, and/or SCV. Therefore, it may be better to use the safe limit of the change in SCV per 1-mm height to calculate the shortening distance, which is relative to the SCV itself, rather than the shortening distance relative to the mean body height of the involved vertebrae.

The main limitation of the present study was that the data were based on a goat model, and the translational value for humans may be limited. However, this study revealed a strong correlation between SCV and SCI due to spinal shortening, and the same phenomenon may occur in the human spinal cord. In the future, to confirm the results of this animal experimental study, a series of severe spinal deformity cases need to be reviewed in order to elucidate the safe limit for shortening in humans.

## Conclusion

The shortening distance strongly correlated with the d-value (post-pre) of SCV per 1-mm height of the osteotomy segment, and showed a good correlation with the T10 body height. The maximum value of the change in SCV per 1-mm height was based on the osteotomy segment, and its safe limit was found to be 142.87 ± 0.59 mm^3^. Thus, our results indicate that the safe limit for shortening can be calculated by using the change in SCV per 1-mm height, and this may be helpful for reducing the risk of neurologic complications in VCR.
